# Negative Test, Persistent Avoidance: Real-World Outcomes of Quinolone De-Labeling

**DOI:** 10.3390/jcm15114334

**Published:** 2026-06-03

**Authors:** Kutay Kırdök, Ceyda Tunakan Dalgıç, Ragıp Fatih Kural, Züleyha Galata, Ümitcan Ateş, Türkan Dizdar Canbaz, Ecem Ay, Eda Aslan, Meryem İrem Toksoy Şentürk, Seda Karaaslan Yetemen, Reyhan Gümüşburun, Emine Nihal Mete Gökmen, Aytül Zerrin Sin

**Affiliations:** Division of Immunology and Allergy, Department of Internal Medicine, Faculty of Medicine, Ege University, 35100 İzmir, Türkiye; kirdokkutay@gmail.com (K.K.); fatih_k_123@hotmail.com (R.F.K.); zuleyhagalata61@gmail.com (Z.G.); umitcanates@gmail.com (Ü.A.); turkan88dizdar@gmail.com (T.D.C.); ecem.ay@live.com (E.A.); edaarslan_91@hotmail.com (E.A.); irem.toksoy@yahoo.com (M.İ.T.Ş.); seda.karaaslan95@gmail.com (S.K.Y.); reyhangumusburun@gmail.com (R.G.); enihalmete@yahoo.com.tr (E.N.M.G.); aytulsin@yahoo.com (A.Z.S.)

**Keywords:** de-labeling, drug provocation test, quinolone hypersensitivity, real-world outcomes, skin test

## Abstract

**Background/Objectives**: Quinolones are among the most frequently implicated antibiotics in immediate hypersensitivity reactions, yet diagnostic evaluation remains challenging and the real-world behaviour of patients after de-labeling has received little attention. We examined the diagnostic work-up, outcomes, and post-de-labeling drug-use behaviour of patients with suspected quinolone hypersensitivity. **Methods**: This single-centre ambispective study included 29 patients evaluated between January 2020 and April 2024. Clinical, demographic, skin test, and drug provocation test (DPT) data were extracted retrospectively. Patients in whom allergy had been excluded or a safe alternative identified were contacted by structured telephone interview between May and June 2025. **Results**: Anaphylaxis was the most common index reaction (42.9%), and ciprofloxacin was the most frequently implicated agent (57.1% of drug instances). Skin tests were positive in 84.2% of cases, but the positive predictive value in the DPT-tested subset was only 20.0%. Of the 12 reached patients, 11 had quinolone allergy excluded; among these, only 4 (36.4%) had reused the de-labeled quinolone, while 7 (63.6%) had avoided it, including 4 who declined it when clinically indicated. **Conclusions**: Quinolone skin tests show limited specificity, and successful de-labeling does not guarantee real-world drug use. Patient anxiety and counselling are central to effective management.

## 1. Introduction

Quinolones are among the most widely prescribed antimicrobials worldwide, owing to their broad spectrum of activity [1,2]. With rising clinical use, the incidence of hypersensitivity reactions to this drug class has also increased [3]. A recent European Academy of Allergy and Clinical Immunology (EAACI) position paper has underscored that, after beta-lactams, quinolones are the second leading antibiotic class responsible for adverse drug reactions [4]. Because these reactions frequently present as life-threatening immediate hypersensitivity events such as anaphylaxis, the appropriate management of quinolone hypersensitivity is of critical importance for both patient safety and rational antibiotic stewardship [3,4,5].

Alongside the classical immunoglobulin E (IgE)-mediated pathway, non-IgE-mediated mechanisms triggered by activation of the Mas-related G-protein coupled receptor member X2 (MRGPRX2) on mast cells play a central role in the pathogenesis of quinolone hypersensitivity [4]. The lack of a routine diagnostic test capable of reliably distinguishing between these two mechanisms, together with the limited sensitivity of advanced methods such as the Basophil Activation Test (BAT) for MRGPRX2-driven events, contributes to considerable uncertainty in clinical management [6,7,8]. Drug provocation tests (DPTs), despite their inherent risk, therefore, remain the diagnostic gold standard [3,4].

Beyond the diagnostic challenges, the management of patients carrying a drug allergy label remains a persistent problem in clinical practice [9,10]. Unnecessary allergy labels drive the use of costly and broad-spectrum alternative antibiotics, contributing both to antimicrobial resistance and to additional economic burden on healthcare systems [11,12]. Yet, whether patients actually reuse a quinolone in real world once allergy has been excluded by provocation testing—and how behavioural barriers influence the de-labeling process—has received surprisingly little attention in the literature [13,14].

Most evidence on post-de-labeling outcomes comes from beta-lactam allergy cohorts, where 60–80% of patients have been shown to safely reuse the implicated antibiotic on long-term follow-up, with severe reactions occurring rarely [15,16,17]. Beyond establishing safety, these programmes have been associated with reduced use of broad-spectrum alternatives—including fluoroquinolones—and with measurable benefits for antimicrobial stewardship [18]. Despite these favourable outcomes, however, a substantial proportion of patients (10–25%) continue to avoid the de-labeled antibiotic in real-world practice, often because of persistent fear of reaction or physician reluctance [19]. Comparable data for quinolones remain remarkably scarce: while the diagnostic performance of skin and provocation testing in quinolone hypersensitivity has been characterised in several studies, systematic evaluations of what happens to patients after diagnostic de-labeling—whether they actually reuse the drug, and how anxiety or counselling shape that decision—have, to our knowledge, not been reported.

The aim of this study was to evaluate a cohort of 29 patients with suspected quinolone hypersensitivity, focusing on (i) their clinical characteristics and diagnostic work-up findings, and (ii) how negative diagnostic results translated into real-world drug use and eventual de-labeling success.

## 2. Materials and Methods

### 2.1. Study Design and Participants

**Figure 1:**
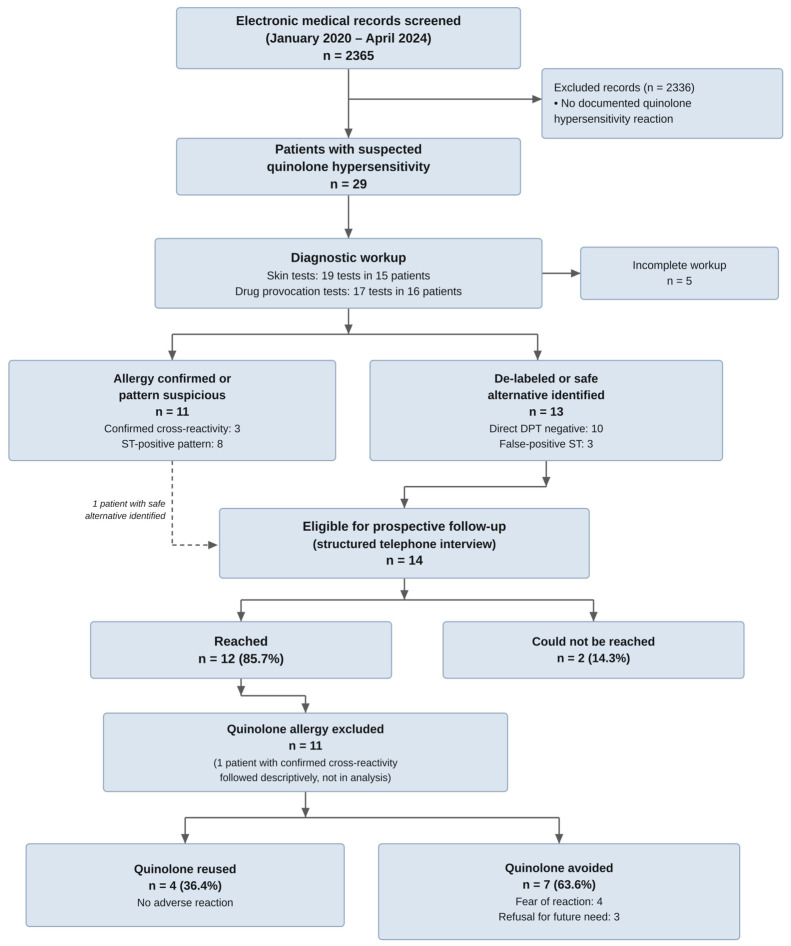
Flow diagram of patient inclusion, diagnostic work-up, and prospective follow-up. Of the 14 patients eligible for follow-up, 12 (85.7%) were successfully contacted by structured telephone interview. The eligible cohort comprised 13 patients in whom quinolone allergy was excluded and 1 patient with confirmed cross-reactivity in whom a safe alternative quinolone was identified. DPT, drug provocation test; ST, skin test.

The retrospective component consisted of a chart review of 2365 electronic medical records from patients who presented to our clinic between January 2020 and April 2024. Patients were included if they were aged 18 years or older and had a documented history of an immediate hypersensitivity reaction temporally associated with quinolone intake. Patients with non-allergic adverse drug reactions known to occur with quinolones—including gastrointestinal symptoms (nausea, vomiting, diarrhoea, abdominal pain, loss of appetite), neuropsychiatric effects (headache, dizziness, insomnia, anxiety, depression, hallucinations), musculoskeletal complaints (tendinitis, myalgia, arthralgia), peripheral neuropathy, QT prolongation, photosensitivity, hepatic enzyme elevation, leukopenia, and tachycardia—were excluded. Twenty-nine patients meeting these criteria were identified and included in the study. For these patients, demographic characteristics, index reaction features, and diagnostic work-up results were extracted from clinical records. The prospective component, conducted between May and June 2025, consisted of a structured telephone interview with all patients in whom quinolone hypersensitivity had been excluded or for whom a safe alternative quinolone had been identified during the diagnostic evaluation. A flow chart of patient inclusion is shown in Figure 1.

The retrospective component of the study, which involved the review of routinely collected, de-identified clinical data, was approved by the Medical Research Ethics Committee of Ege University Faculty of Medicine (Decision No: 2025-480125-3.1T/82, date of approval: 24 March 2025). Retrospective studies based on the review of file and image records—which do not involve direct intervention on the patient—are conducted under Ethics Committee approval without requiring individual informed consent. The prospective component consisted of a structured telephone interview to collect patient-reported information about subsequent drug use, without any new diagnostic or therapeutic intervention. As the interview was conducted exclusively by telephone, and patients could not be reached by reliable postal correspondence or feasibly required to attend the clinic in person solely for the purpose of providing written consent, verbal informed consent was obtained at the beginning of each interview, after the investigator had explained the study’s purpose, procedures, and voluntary nature, and was documented in the research record. All procedures were conducted in accordance with the principles of the Declaration of Helsinki.

### 2.2. Clinical Data and Index Reaction Characteristics

Clinical and demographic data were collected using a standardized data extraction form. Recorded variables included age, sex, history of atopy, and relevant comorbidities, as well as the suspected quinolone agent and the clinical manifestations observed during the hypersensitivity reaction. The organs and systems involved were documented in detail.

The severity of reactions was graded according to Brown’s classification of acute systemic hypersensitivity reactions, corresponding to mild (Grade 1; cutaneous and subcutaneous manifestations only), moderate (Grade 2; features suggesting respiratory, cardiovascular, or gastrointestinal involvement), and severe (Grade 3; hypoxia, hypotension, or neurological compromise) reactions [20].

In addition, the time interval between quinolone intake and onset of symptoms was recorded, and the duration between the index reaction and the allergy work-up was analyzed to evaluate potential factors affecting diagnostic performance.

### 2.3. Drug Allergy Work-Up

All diagnostic procedures were carried out by an experienced allergist, using concentrations previously reported as non-irritant in the literature [4,7]. Non-irritant thresholds refer to drug concentrations that do not elicit non-specific skin reactions in non-allergic individuals; concentrations exceeding these thresholds may produce false-positive results and are therefore avoided in the diagnostic work-up. To mitigate the risk of immediate hypersensitivity reactions during the work-up, all tests were performed in a hospital setting equipped with emergency resuscitation equipment and under appropriate clinical monitoring. Five patients did not complete the planned diagnostic evaluation after the initial consultation; these patients were retained in the cohort for demographic and clinical characterization but were excluded from diagnostic performance and prospective follow-up analyses.

#### 2.3.1. Skin Tests

Skin test (ST) concentrations were selected based on previously reported non-irritant concentrations described in the literature and in accordance with the EAACI position paper on quinolone hypersensitivity [4,21,22]. STs were applied on the volar surface of the forearm using histamine (ApiPrick; ASAC Pharmaceutical, Alicante, Spain) and saline as positive and negative controls, respectively. Test results were evaluated after 20 min, and a wheal ≥ 3 mm larger than the negative control accompanied by erythema was considered positive [7].

Detailed concentrations and dilution schemes used for each quinolone are summarized in Table 1.

#### 2.3.2. Drug Provocation Tests

Drug provocation tests (DPTs) were performed to confirm or exclude quinolone hypersensitivity and to identify a safe alternative quinolone when clinically indicated. The decision to perform DPT was made on an individualized basis by the treating allergist in consultation with the patient.

Single-blind, placebo-controlled protocols were used. Testing began with administration of a placebo preparation followed by stepwise incremental doses of the active drug at 30 min intervals until the full therapeutic dose was achieved or objective clinical findings occurred. The specific cumulative dosing schedules used for each tested quinolone are detailed in Table 1. In patients with a positive skin test, the decision to perform DPT was made on an individualised basis, balancing the potential diagnostic benefit against the risk of provoking a severe reaction. Patients with a history of severe anaphylaxis at the index reaction, those for whom a tolerated alternative quinolone had already been identified, and those who declined the procedure did not undergo provocation testing. On this basis, drug provocation testing was performed in 4 of the 12 patients with a positive skin test.

A test was considered positive when objective signs consistent with the index reaction (e.g., urticaria, angioedema, respiratory symptoms, or systemic reactions) were observed. Subjective complaints in the absence of objective clinical findings were not considered sufficient to define test positivity.

All provocation procedures were performed in a hospital setting with emergency resuscitation equipment readily available, and patients were monitored for at least three hours after the final dose to detect immediate hypersensitivity reactions.

### 2.4. Prospective Follow-Up and Assessment of Real-World Drug Use

During the prospective phase, patients in whom quinolone hypersensitivity had been excluded or for whom a safe alternative quinolone had been identified were contacted through a structured telephone interview conducted by a trained physician investigator. The interview followed a standardised questionnaire developed by the study team and covered the following items:

(i) Whether the patient had received a clinical indication for quinolone use during the follow-up period;
(ii) Whether the identified safe quinolone had been actually reused, and if so, on how many occasions, the indication, and the duration of treatment;
(iii) The occurrence, timing (after the first or subsequent doses), clinical features, and severity of any adverse reaction experienced during reuse;
(iv) For patients who had not reused the drug despite a negative diagnostic evaluation, the reason for non-use (e.g., fear of reaction, refusal despite physician recommendation, alternative antibiotic prescribed by another clinician, no clinical need during follow-up); and
(v) The patient’s stated willingness to reuse the drug in a future clinical situation requiring it.

Interviews lasted approximately 10–15 min. Each patient was contacted on up to three separate occasions before being classified as not reachable. The number of patients who could not be contacted was documented.

### 2.5. Statistical Analysis

Statistical analysis was performed using SPSS version 26.0 (IBM Corp., Armonk, NY, USA). Given the exploratory design and limited sample size, analyses were primarily descriptive. Continuous variables are presented as mean ± standard deviation (SD) or median with interquartile range (IQR), as appropriate. Categorical variables are expressed as absolute numbers and percentages (*n*, %). Diagnostic outcomes and real-world follow-up data were summarized using descriptive statistics.

During the preparation of this manuscript the authors used Claude (Anthropic version 4.7) for English language translation manuscript formatting assistance and figure design support. The authors have reviewed and edited the output and take full responsibility for the content of this publication.

## 3. Results

### 3.1. Patient Characteristics and Clinical Background

A total of 29 patients were evaluated (mean age 46.4 ± 16.2 years; 20 female, 69.0%). Concomitant non-quinolone drug hypersensitivity was documented in 21 patients (72.4%), most frequently involving beta-lactam antibiotics (16/29, 55.2%) and NSAIDs (8/29, 27.6%). Documented clinical presentation of the index reaction was available for 28 patients; anaphylaxis was the most common phenotype (12/28, 42.9%), followed by urticaria (8/28, 28.6%). According to Brown’s classification, most anaphylactic reactions were graded as moderate (Grade 2; 10/12, 83.3%), while two patients (2/12, 16.7%) experienced severe (Grade 3) reactions with hypotension or syncope. Detailed demographic and clinical characteristics are summarized in Table 2.

### 3.2. Implicated Quinolones and Reaction Phenotypes

**Figure 2:**
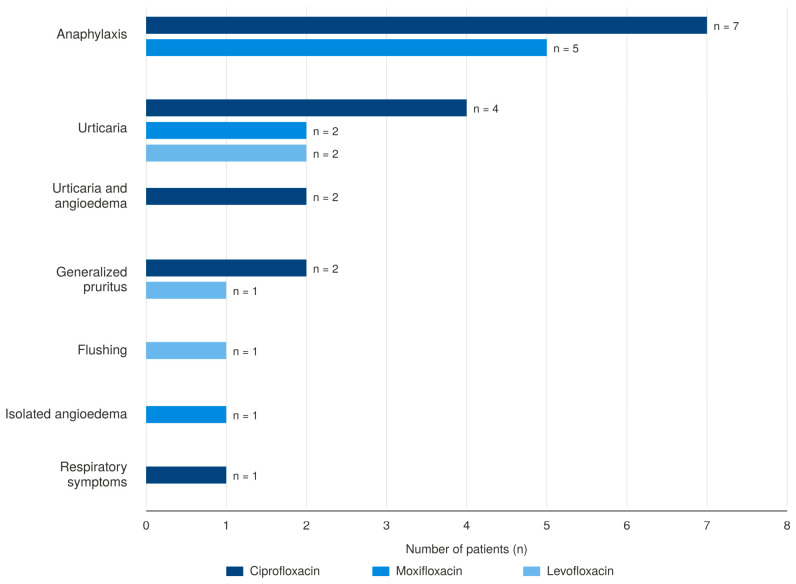
Distribution of culprit quinolones according to clinical presentation of the index reaction. Bars represent the number of patients per phenotype, grouped by implicated drug. Data are shown for the 28 patients with a documented index reaction. One additional patient (P11), who reported two simultaneously suspected culprits (moxifloxacin and levofloxacin) but for whom the clinical presentation of the index reaction was not documented, is not included in this figure.

One patient reported two simultaneously suspected culprits (moxifloxacin and levofloxacin), resulting in a total of 28 implicated drug instances across the 28 patients. Ciprofloxacin was the most frequently implicated agent, accounting for 16 of 28 drug instances (57.1%), followed by moxifloxacin (8/28, 28.6%) and levofloxacin (4/28, 14.3%). Ciprofloxacin was also responsible for the majority of anaphylaxis cases (7/12, 58.3%), whereas no anaphylaxis was attributed to levofloxacin, which was more frequently associated with milder cutaneous manifestations such as flushing and pruritus. The distribution of implicated agents according to clinical presentation is shown in Figure 2.

### 3.3. Diagnostic Work-Up: Skin Testing and Drug Provocation Tests

**Figure 3:**
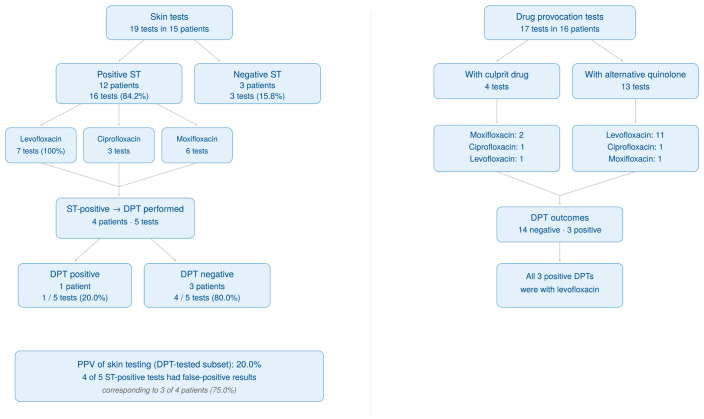
Results of the diagnostic work-up. (**Left**) panel: skin test (ST) outcomes—16 of 19 ST were positive (84.2%), and 4 ST-positive patients subsequently underwent drug provocation test (DPT), of whom only 1 had an objective reaction. (**Right**) panel: DPT outcomes—17 DPTs were performed in 16 patients, with 3 positive results, all occurring with levofloxacin. None of the 3 ST-negative patients underwent DPT. DPT, drug provocation test; PPV, positive predictive value; ST, skin test.

Of the 29 included patients, 24 completed the diagnostic work-up. The remaining 5 did not undergo further testing after the initial consultation and were classified as having an incomplete work-up. A total of 19 skin tests were performed in 15 patients, of which 16 (84.2%) were positive; this corresponded to 12 patients (80.0% of skin-tested patients) with at least one positive skin test result. All STs performed with levofloxacin were positive (7/7, 100%). Seventeen DPTs were subsequently conducted in 16 patients, resulting in three objective reactions (3/17, 17.6%), all occurring with levofloxacin. Four patients (P1, P2, P11, and P12) underwent both skin testing and drug provocation testing, providing direct correlation between the two diagnostic modalities. Detailed diagnostic outcomes are illustrated in Figure 3.

Among the four skin test-positive patients who subsequently underwent DPT, five provocation tests were performed; one was positive (1/5 tests, 20.0%; corresponding to 1/4 patients, 25.0%) and the remaining four tests (in three patients) were negative. The positive predictive value of skin testing in the DPT-tested subset was therefore 20.0% on a per-test basis. None of the skin test-negative patients underwent DPT, so the negative predictive value could not be formally assessed. The individual diagnostic patterns observed in these patients, including the combinations of skin test positivity and provocation test outcomes with different quinolones, are summarized in Table 3.

### 3.4. Real-World Outcomes and Follow-Up

Of the 14 patients eligible for prospective follow-up (quinolone hypersensitivity excluded or a safe alternative identified), 12 (85.7%) were successfully contacted and 2 (14.3%) could not be reached. One of these 12 patients (P12) had been classified as having confirmed cross-reactivity to levofloxacin during the diagnostic work-up; she was inadvertently prescribed ciprofloxacin by another physician during a subsequent infection and tolerated it uneventfully, despite the fact that ciprofloxacin had not been formally tested in our clinic. This observation is reported descriptively and is not included in the de-labeling success analysis. Among the remaining 11 patients in whom quinolone hypersensitivity had been excluded, 4 (36.4%) had reused the quinolone that was identified as safe during the diagnostic work-up and tolerated it without any adverse reaction.

Seven of these 11 patients (63.6%) had not reused the identified quinolone. Of these, 4 patients (36.4%) had required antibiotic therapy during the follow-up period but had actively declined the recommended quinolone, while 3 patients (27.3%) had not required antibiotic therapy during follow-up but stated that they would refuse the drug even if it were clinically needed in the future. The individual real-world outcomes are summarized in Table 3.

## 4. Discussion

This single-centre study examined the clinical features, diagnostic test results, and post-de-labeling real-world outcomes of patients referred for evaluation of suspected quinolone hypersensitivity. What distinguishes our work from previously published cohorts is that, beyond reporting diagnostic findings, we also assessed whether patients actually reused the drug in everyday clinical life once their allergy had been excluded. Our findings point to a clear mismatch between skin test and drug provocation test results in the evaluation of quinolone hypersensitivity, and reveal that a substantial proportion of patients continue to avoid the drug despite negative diagnostic testing.

Women are known to report drug-related adverse reactions and to seek medical attention more frequently than men [1], and in keeping with this, 69.0% of our patients were female [8,23]. Although data specific to quinolone hypersensitivity are still scarce, the higher prevalence in women has been attributed to a combination of hormonal and immunological differences, X chromosome-linked genetic factors, greater cumulative drug exposure, and more frequent use of healthcare services [24,25,26]. Similarly, in older patients, the combination of frequent quinolone prescribing, comorbidities, and polypharmacy is thought to account for the higher reporting rates of both adverse drug reactions and allergic reactions [23,27,28]. Of note, our cohort was concentrated in the middle-age range, a pattern that is itself meaningful given that accumulated drug exposure and prior reaction history can shape subsequent treatment decisions.

A history of chronic spontaneous urticaria (CSU) was documented in 13.8% of our patients, and 10.3% had asthma. Beyond the classical IgE-mediated pathway, quinolones are also known to drive non-specific mast cell activation through the MRGPRX2 receptor on mast cells [2]. This mechanism may help explain the false-positive skin test results encountered in conditions of heightened mast cell reactivity such as CSU [6]. Coexisting airway disease adds a further layer of complexity to clinical interpretation: in some cases, respiratory symptoms may stem from an underlying asthma exacerbation, and dyspnea occurring in close temporal proximity to drug intake can be mistaken for a hypersensitivity reaction [29]. For these reasons, the presence of CSU or asthma warrants careful consideration when assessing a suspected quinolone hypersensitivity.

In our cohort, 55.2% of patients had a documented history of beta-lactam hypersensitivity and 10.3% reported hypersensitivity to macrolides, indicating that allergy labels spanning multiple antibiotic classes are common in this population. Previous reports have described an increased risk of quinolone hypersensitivity in patients with a pre-existing beta-lactam allergy [5,30]. Whether this association reflects a genuine immunological predisposition or simply the fact that quinolones are more frequently prescribed to beta-lactam-allergic patients, however, remains unclear.

Anaphylaxis accounted for the majority of referrals to our clinic, followed by urticaria and other cutaneous presentations. In line with previous reports, our results confirm that quinolone hypersensitivity can manifest across a broad clinical spectrum [3]. Interestingly, however, a subset of our patients presented with less specific early features such as isolated generalised pruritus or flushing. This suggests that cutaneous manifestations of quinolone hypersensitivity may extend beyond the classical urticaria and angioedema, and that in some cases the reaction may begin with prodromal or atypical symptoms. Clinically, this implies that even isolated pruritus or flushing occurring after quinolone exposure deserves to be considered within the differential diagnosis of drug-related hypersensitivity.

Ciprofloxacin emerged as the most frequently implicated quinolone in our cohort, accounting for 57.1% of index reactions and a substantial share of anaphylaxis cases. Levofloxacin, by contrast, was not associated with any anaphylactic event and was predominantly linked to milder cutaneous reactions. Quinolones are known to trigger IgE-independent mast cell activation via the MRGPRX2 receptor, a mechanism that may particularly underlie cutaneous manifestations such as pruritus, flushing, and urticaria [2,31,32]. Levofloxacin, however, has been suggested to possess a lower intrinsic capacity to activate mast cells in an IgE-independent manner compared with ciprofloxacin and moxifloxacin [6], which could offer a plausible mechanistic explanation for its predominant association with mild cutaneous symptoms in clinical practice.

Our results also highlight a clear discrepancy between skin testing and drug provocation testing in the diagnostic evaluation of quinolone hypersensitivity. Although skin tests were positive in 84.2% (16/19) of cases, confirmation by DPT was limited: for safety reasons, most skin test-positive patients were spared from provocation and directed to alternative agents, and among the four patients who did undergo DPT after a positive skin test, only one reacted. These findings suggest that the positive predictive value of quinolone skin testing may be modest, and that false-positive results are likely to represent a meaningful clinical problem in everyday practice [5].

A particularly striking pattern was observed between diagnostic test results and real-world tolerance in one of our patients: following an index reaction attributed to moxifloxacin, the patient subsequently showed a positive provocation test to levofloxacin, yet later tolerated ciprofloxacin uneventfully when it was prescribed elsewhere during a subsequent infection. While this observation confirms cross-reactivity between moxifloxacin and levofloxacin, it also suggests that ciprofloxacin may not share the same cross-reactivity pattern with these two agents. Taken together, this case indicates that cross-reactivity in quinolone hypersensitivity may not always follow a class-wide pattern and reinforces the importance of evaluating each alternative drug on an individual basis rather than relying on class-level assumptions.

When real-world data from patients whose quinolone allergy had been excluded by provocation testing were examined, diagnostic results were not consistently mirrored in subsequent drug-use behaviour. While some patients later took the recommended quinolone uneventfully during a new infection, a considerable proportion avoided the drug altogether. Specifically, 36.4% (4/11) of patients refused to use the drug because of persistent fear of reaction despite negative testing, while a further 27.3% (3/11) had not required antibiotic therapy during follow-up but stated that they would refuse the drug even if a clinical need were to arise. These findings show that even relatively mild Grade 1–2 reactions can leave a lasting anxiety in patients, and that avoidance behaviour may persist despite the medical reassurance provided by a negative provocation test.

The psychological dimension of post-de-labeling avoidance warrants particular attention. In our clinic, every patient with a negative provocation test receives both verbal counselling—explaining that the risk of a future hypersensitivity reaction is comparable to that of the general population—and a written documentation card stating that quinolone hypersensitivity had been excluded or that a safe alternative quinolone had been identified and could be used safely. Despite this structured reassurance, more than half of the de-labeled patients continued to avoid the drug. Notably, when asked directly during the follow-up interview, none of the patients attributed their avoidance to the recommendation of another physician; several reported that the drug had even been re-prescribed by a treating clinician during a subsequent infection but had been refused by the patient themselves. This pattern suggests that the persistence of avoidance behaviour in our cohort was driven primarily by patient-level factors—most prominently residual fear and altered perception of personal risk—rather than by physician reluctance to re-prescribe. These observations are consistent with the broader drug allergy literature, in which negative diagnostic results do not consistently translate into restored confidence in the drug, and the experience of the initial hypersensitivity reaction itself shapes long-term risk perception independently of subsequent reassurance [13,14,19].

A similar pattern has been described in real-world studies of proton pump inhibitors, where alternative agents shown to be safe by DPT were ultimately not used by 55.6% of patients, owing to fear of reaction or physician reluctance—emphasising that medical success on paper does not always translate into real-world drug use [9]. These observations argue that successful drug allergy management depends not only on diagnostic accuracy but equally on addressing patient anxieties and providing appropriate counselling, if negative test results are to translate into actual drug use.

### Limitations

This study has several limitations. The single-centre, ambispective design and the relatively small sample size (*n* = 29) inevitably limit the generalisability of our findings; the limited number of events also precludes the use of multivariate or predictive statistical modelling, and the analyses presented here are, therefore, deliberately descriptive. Given these constraints, the findings of this study should be regarded as hypothesis-generating rather than definitive. The reported proportions—particularly those derived from small subgroups, such as the four patients who underwent both skin and provocation testing—should be interpreted with corresponding caution and cannot be reliably extrapolated to the broader population of patients with suspected quinolone hypersensitivity. A further caveat concerns the way diagnostic testing was delivered: rather than following a pre-specified protocol, decisions were made on a case-by-case basis, which opens the door to selection bias. As a result, both the diagnostic performance estimates and the patient-reported reasons for post-de-labeling avoidance—the latter additionally subject to recall bias—should be interpreted with due caution. In particular, among the eight skin test-positive patients in whom drug provocation was not attempted, the proportion of false-positive results might well have been even higher than in the tested subset, and the positive predictive value reported here should be read with that in mind. For the same reason, the three skin test-negative patients who did not undergo provocation preclude any reliable estimate of the negative predictive value.

Five additional patients were unable to complete the planned work-up after the initial consultation and were therefore excluded from both the diagnostic performance analysis and the prospective follow-up. Another point that warrants caution is the uniformly positive skin test results obtained with levofloxacin, which raise the possibility that the concentrations we used may have crossed the irritant threshold. Non-irritant concentrations for fluoroquinolones remain a matter of ongoing debate, and we cannot entirely rule out a contribution of irritant effects to this finding. The prospective follow-up relied on structured telephone interviews, which carries the usual risks of recall and response bias inherent to self-reported data. Finally, because we were unable to perform molecular analyses of MRGPRX2, our mechanistic interpretations remain grounded in clinical observation rather than in direct laboratory confirmation.

## 5. Conclusions

In this exploratory single-centre cohort, skin testing in suspected quinolone hypersensitivity showed considerable discordance with drug provocation test results: although limited to a small subset of skin test-positive patients who underwent provocation testing, most of these patients tolerated drug provocation without reaction. Although limited by the small number of patients undergoing both tests, these findings support caution in interpreting a positive quinolone skin test as definitive evidence of clinical reactivity, particularly in the absence of confirmatory provocation testing. Drug provocation testing therefore remains the cornerstone of definitive diagnosis and safe de-labeling. The most striking finding of our study, however, is that a substantial proportion of patients whose allergy had been formally excluded continued to avoid the drug in real-world practice; nearly two-thirds reported persistent avoidance overall, and more than one-third declined the drug even when antibiotic therapy was clinically indicated. This observation underlines that patient education, and the building of trust are indispensable components of effective drug allergy management. Allergists, prescribing physicians, and clinical pharmacists should therefore approach de-labeling not as a diagnostic endpoint but as the starting point of a longer patient-centred process, in which structured counselling, clear documentation of test results, and follow-up consultations may be needed to bridge the gap between diagnostic exclusion and actual real-world drug use. Confirmation of these observations in larger multicentre cohorts will be essential to establish their generalisability and to identify factors that may predict persistent avoidance after diagnostic de-labeling.

## Figures and Tables

**Table 1:** Concentrations and protocols used for skin testing and drug provocation testing of quinolones.

Drug	Available Formulation	Prick Test Concentration	Intradermal Starting Dilution	Maximum Intradermal Concentration	DPT Cumulative Dose Protocol
Ciprofloxacin	2 mg/mL	1/300 dilution	1/300	1/300	5 mg → 50 mg → 100 mg → 345 mg (cumulative 500 mg)
Moxifloxacin	1.6 mg/mL	1/100 dilution	1/1000	1/100	4 mg → 40 mg → 80 mg → 276 mg (cumulative 400 mg)
Levofloxacin	5 mg/mL	5 mg/mL or diluted protocol in anaphylaxis cases	1/2000	1/200	5 mg → 50 mg → 100 mg → 345 mg (cumulative 500 mg)

Concentrations are based on previously published non-irritant thresholds for fluoroquinolones [4,7]. Skin tests were performed sequentially: skin prick tests followed by intradermal tests at increasing dilutions, with each step interpreted before proceeding to the next. Drug provocation tests were performed using single-blind, placebo-controlled protocols with cumulative oral dosing. Abbreviations: DPT, drug provocation test.

**Table 2:** Baseline demographic and clinical characteristics of the study cohort.

Variable	Value (*n*, %)
**Demographics**	
Age, years (mean ± SD)	46.4 ± 16.2
Female	20 (69.0%)
**Allergic Background**	
Chronic Spontaneous Urticaria	4 (13.8%)
Asthma	3 (10.3%)
**Non-quinolone drug hypersensitivity** ^a^	
Beta-lactam antibiotics	16 (55.2%)
NSAIDs	8 (27.6%)
Macrolides	3 (10.3%)
Paracetamol	2 (6.9%)
Metronidazole	2 (6.9%)
Proton pump inhibitor	1 (3.4%)
Antifungals	1 (3.4%)
Aminoglycoside (gentamicin)	1 (3.4%)
**Clinical Presentation** ^b^	
Anaphylaxis	12 (42.9%)
Urticaria	8 (28.6%)
Generalized Pruritus	3 (10.7%)
Angioedema + Urticaria	2 (7.1%)
Flushing	1 (3.6%)
Isolated Angioedema	1 (3.6%)
Respiratory symptoms	1 (3.6%)

Data are presented as *n* (%) unless otherwise indicated. ^a^ Some patients had hypersensitivity to more than one non-quinolone drug class; the total number of events therefore exceeds the number of patients. ^b^ Clinical presentation data were unavailable for 1 patient; percentages calculated from *n* = 28. Abbreviations: NSAID, non-steroidal anti-inflammatory drug; SD, standard deviation.

**Table 3:** Individual clinical features, diagnostic test results, and real-world outcomes for each patient.

Patient	Age/Sex	Index Reaction	Culprit Quinolone	ST Result	DPT Result	Diagnostic Classification	Real-World Use	Reason for Non Use
P1	33/M	AN (G2)	M	M (+), L (+)	M (−)	False-positive ST, de-labeled	Avoided	Fear of reaction
P2	46/M	AN (G2)	M	C (+), L (+)	C (−), L (−)	False-positive ST, de-labeled	Avoided	Fear of reaction
P3	29/F	AN (G2)	C	N.A.	L (−)	De-labeled	Avoided	Fear of reaction
P4	52/M	AN (G2)	C	N.A.	L (−)	De-labeled	Avoided	Fear of reaction
P5	59/M	U + AE	C	N.A.	L (−)	De-labeled	Not required	Refusal despite potential need
P6	19/F	AN (G2)	C	N.A.	L (−)	De-labeled	Not required	Refusal despite potential need
P7	30/F	U	C	N.A.	M (−)	De-labeled	Not required	Refusal despite potential need
P8	32/F	AN (G2)	C	N.A.	L (−)	De-labeled	Used	-
P9	44/F	AN (G2)	C	N.A.	L (−)	De-labeled	Used	-
P10	45/M	AE	M	N.A.	M (−)	De-labeled	Used	-
P11	51/F	-	L, M	L (+), M (+)	L (−)	False-positive ST, de-labeled	Used	-
P12	34/F	AN (G2)	M	L (+)	L (+)	Confirmed cross-reactivity	Used (C)	-
P13	54/F	GP	C	N.A.	L (−)	De-labeled	No response	-
P14	57/M	U	C	N.A.	C (−)	De-labeled	No response	-
P15	55/F	U + AE	C	N.A.	L (+)	Confirmed cross-reactivity	-	-
P16	55/F	U	C	N.A.	L (+)	Confirmed cross-reactivity	-	-
P17	67/F	RS	C	M (+)	N.A.	ST-positive pattern	-	-
P18	46/F	U	M	C (+)	N.A.	ST-positive pattern	-	-
P19	34/F	F	L	L (+)	N.A.	ST-positive pattern	-	-
P20	33/M	GP	L	L (+)	N.A.	ST-positive pattern	-	-
P21	70/F	U	M	M (+)	N.A.	ST-positive pattern	-	-
P22	25/F	AN (G2)	C	M (+)	N.A.	ST-positive pattern	-	-
P23	49/M	AN (G3)	M	C (+), L (+)	N.A.	ST-positive pattern	-	-
P24	61/M	U	L	M (+)	N.A.	ST-positive pattern	-	-
P25	81/F	GP	C	N.A.	N.A.	Incomplete work-up	-	-
P26	53/F	U	C	C (−)	N.A.	Incomplete work-up	-	-
P27	32/M	AN (G2)	M	C (−)	N.A.	Incomplete work-up	-	-
P28	26/F	AN (G3)	C	N.A.	N.A.	Incomplete work-up	-	-
P29	61/F	U	L	M (−)	N.A.	Incomplete work-up	-	-

Patients are listed in numerical order (P1–P29). Skin test and drug provocation test results are reported per drug. Plus and minus signs in parentheses indicate test outcome (positive or negative). N.A., test not performed. Index reaction details were not documented for one patient (P11). Two patients with confirmed cross-reactivity (P15 and P16) were not eligible for prospective follow-up because no safe alternative quinolone had been formally identified at the time of evaluation. One additional patient (P12) classified as having confirmed cross-reactivity tolerated ciprofloxacin during follow-up, although ciprofloxacin had not been formally tested in our clinic; this observation is reported descriptively and is not included in the de-labeling success analysis. Abbreviations: In the Age/Sex column, M denotes male and F denotes female. AE, angioedema; AN, anaphylaxis; C, ciprofloxacin; DPT, drug provocation test; F, flushing; G2, Brown grade 2 (moderate); G3, Brown grade 3 (severe); GP, generalised pruritus; L, levofloxacin; M, moxifloxacin; N.A., not available; RS, respiratory symptoms; ST, skin test; U, urticaria.

## Data Availability

The data supporting the findings of this study are available from the corresponding author upon reasonable request. The data are not publicly available due to privacy and ethical restrictions related to patient confidentiality.

## Abbreviations

The following abbreviations are used in this manuscript:

ADR	Adverse drug reaction
BAT	Basophil activation test
CSU	Chronic spontaneous urticaria
DPT	Drug provocation test
EAACI	European Academy of Allergy and Clinical Immunology
ID	Intradermal test
IgE	Immunoglobulin E
MRGPRX2	Mast-related G-protein coupled receptor member X2
NPV	Negative predictive value
NSAID	Non-steroidal anti-inflammatory drug
PPI	Proton pump inhibitor
PPV	Positive predictive value
SD	Standard deviation
SPT	Skin prick test
ST	Skin test
